# Thoracoscopic surgery

**DOI:** 10.4103/0972-9941.38904

**Published:** 2007

**Authors:** H. S. Bhanushali

**Affiliations:** Department of Surgery, Kaushalya Medical Foundation Trust Hospital, Behind Nitin Co Ganeshwadi Panchpakhadi, Thane (West), Thane - 400 601, India

“Mine is yesterday, I know tomorrow.”

*Tibetan book of BC 3500*

I am grateful to the editorial board of Journal of Minimal Access Surgery for requesting me to be the Guest Editor for this special issue on Thoracoscopy. Never did I think that that thoracoscopy will reach such an eminence and level of acceptance worldwide. It is a tribute to surgeons who are involved in performing minimal access surgery who have taken to thoracoscopic technique all over the world, since minimal invasive surgery was started since 1991. We started doing thoracoscopy in 1991.

Thousands of thoracoscopic procedures were performed for the division of pleural adhesions between 1910, (the year the technique was instituted by the Swedish internist Hans Jacobeus) and 1955 (when the technique became obsolete following discovery of anti tuberculous drugs). Thoracoscopy was carried out only under local anesthesia using only primitive instruments and lighting.

Why is it that after several decades of infrequent use thoracoscopic procedures are again gaining importance throughout the world? The use of thoracoscopy has been resumed partly as a result of considerable progress in modern techniques, particularly in the following two areas: 1) Endoscopic instrumentation has been greatly improved; telescopes now have an extremely high optical quality in spite of their very small diameter and 2) Progress in anesthesia has allowed for a wide choice ranging from local anesthesia for outpatients to general anesthesia with endotracheal intubation (single lung anaesthesia). Jacobeus himself believed that thoracoscopy could be used not only to divide pleural adhesions but also perform pleural biopsies for histopathology and early diagnosis of pleural pathology - the commonest one in his times being tuberculosis. In addition to visualization of pleural surfaces, the lung itself can be examined from various angles. In spontaneous pneumothorax minute emphysematous bullous lesions and blebs less than 5 mm in diameter are seen nearly in all patients when the lungs are examined using a high resolution video camera and xenon light. It can therefore be said that no patient with spontaneous pneumothorax has a “normal lung”. The information obtained by thoracoscopy in these patients is superior to that derived by high resolution CT scanning or any other investigative modality short of open thoracotomy.

The entire thoracic cavity is accessible to video assisted thoracoscopy permitting diagnosis of peripheral pulmonary lesions - whether diffuse or localized nodules, chronic empyema and also benign or malignant pathology. Thoracoscopy can easily replace many diagnostic and therapeutic procedures hitherto carried out by open surgery involving a thoracotomy. It is possible to carry out biopsies from diaphragmatic surfaces, pericardium, mediastinal nodes or masses. Sectioning of sympathetic nerves (sympathectomy) can be used for vasomotor syndromes affecting the upper limb. Drainage of empyema, decortication of stage II empyema, coagulation of blebs and excision of bullae in pneumothorax as well as advanced procedures such as esophageal mobilization and resection can be performed by a surgeon accomplished at thoracoscopic surgery.

Thoracoscopy, while allowing full exploration of the thoracic cavity is much less invasive and incapacitating than thoracotomy. Complications are uncommon and occur rarely when the procedure is performed by someone who has mastered the technique. The cost of treatment is much less than thoracotomy because of the shorter period of hospitalization; an average of no more than two to five days. There are no sequelae as long as effective pleural drainage ensures complete pleurodesis. No rehabilitation is required and a normal active life can be resumed within a few days.

Thoracoscopy has not remained the domain of thoracic surgeons but cardiac surgeons too perform pericardiectomy, coronary artery bypass surgery using minimal access thoracic techniques. Also orthopedic surgeons undertake spinal surgeries such as biopsy from spinal lesions, drainage of spinal cold abscesses and complex corrective surgeries for scoliosis.

This special issue has two main goals. Firstly, it highlights that thoracoscopic techniques can be used for myriad of conditions as seen from the various articles appearing in this issue. Secondly, the issue also underscores the point that with adequate training and a thorough understanding of the basic principles of thoracic surgery, even general surgeons can perform basic thoracosocpic procedures. In the hands of accomplished surgeons, sky seems to be the limit for the use of thoracoscopy in thoracic pathology.

